# *In vivo* whole brain microvascular imaging in mice using transcranial 3D Ultrasound Localization Microscopy

**DOI:** 10.1016/j.ebiom.2022.103995

**Published:** 2022-04-20

**Authors:** Oscar Demeulenaere, Adrien Bertolo, Sophie Pezet, Nathalie Ialy-Radio, Bruno Osmanski, Clément Papadacci, Mickael Tanter, Thomas Deffieux, Mathieu Pernot

**Affiliations:** a Physics for Medicine, ESPCI, Inserm, CNRS, Institute of Physics for Medicine Paris, PSL University, ESPCI Paris, 17 rue Moreau, Paris 75012, France; b Iconeus, Paris 75014, France

**Keywords:** Ultrasound localization microscopy, Mouse brain, Vascular atlas, Flow imaging, Azc, Azygos Anterior Cerebral Artery, FWHM, Full Width Half Maximum, IM, Intramuscular, ICP, Iterative Closest Point, MB, Micro-Bubbles, Mca, Medial Cerebral Artery, micro-CT, Micro Computed Tomography, MIP, Maximum Intensity Projection, MR, Magnetic Resonance imaging, Pca, Posterior Cerebral Artery, PPC, Posterior Parietal cortex, PRF, Pulse Repetition Frequency, PSF, Point Spread Function, RF, Radio-Frequency, SNR, Signal to Noise Ratio, Sss, Superior Sagittal Sinus, ULM, Ultrasound Localization Microscopy

## Abstract

**Background:**

Non-invasive high-resolution imaging of the cerebral vascular anatomy and function is key for the study of intracranial aneurysms, stenosis, arteriovenous malformations, and stroke, but also neurological pathologies, such as degenerative diseases. Direct visualization of the microvascular networks in the whole brain remains however challenging *in vivo*.

**Methods:**

In this work, we performed 3D ultrafast ultrasound localization microscopy (ULM) using a 2D ultrasound matrix array and mapped the whole-brain microvasculature and flow at microscopic resolution in C57Bl6 mice *in vivo*.

**Findings:**

We demonstrated that the mouse brain vasculature can be imaged directly through the intact skull at a spatial resolution of 20 µm and over the whole brain depth and at high temporal resolution (750 volumes.s^−1^). Individual microbubbles were tracked to estimate the flow velocities that ranged from 2 mm.s^−1^ in arterioles and venules up to 100 mm.s^−1^ in large vessels. The vascular maps were registered automatically with the Allen atlas in order to extract quantitative vascular parameters such as local flow rates and velocities in regions of interest.

**Interpretation:**

We show the potential of 3D ULM to provide new insights into whole-brain vascular flow in mice models at unprecedented vascular scale for an *in vivo* technique. This technology is highly translational and has the potential to become a major tool for the clinical investigation of the cerebral microcirculation.

**Funding:**

This study was supported by the European Research Council under the European Union's Seventh Framework Program (FP/2007-2013) / ERC Grant Agreement n° 311025 and by the Fondation Bettencourt-Schueller under the program “Physics for Medicine”. We acknowledge the ART (Technological Research Accelerator) biomedical ultrasound program of INSERM.

**Research in context**

**Evidence before this study**

Imaging the vascular networks in the whole brain remains challenging *in vivo*, as it spans multiple spatial scales (from micron-sized capillaries to millimeter-sized vessels) and multiple scales of velocity (from ∼1 mm/s in capillaries to ∼100mm/s in larger vessels). Current imaging modalities are limited by either the spatial resolution (MRI, CT scans, PET) or by the penetration depth (optical imaging). In contrast, Ultrafast Ultrasound Localization Microscopy (ULM) was introduced in 2015 to map the microvascular flows at high spatial resolution (∼10µm) and at depths of several centimeters, much greater than the traditionally frequency-limited imaging depth. This concept has already been applied in-vivo for the mapping of cerebral microvascular flow in 2D at first in rodents and very recently in humans. Nevertheless, cerebral vessels are oriented in all three dimensions and 2D velocity estimates can be significantly underestimated when the vessels are not perfectly aligned to the 2D slice. Several approaches have been proposed to extend ULM to 3D but none of them have been used to image the mouse brain through the intact skull.

**Added value of this study**

We show the potential of 3D ULM to provide new insights into whole-brain vascular flow in mice models at unprecedented vascular scale for an *in vivo* technique. Microvascular flows were imaged directly at a spatial resolution of 20 µm enabling the quantification of diameter, flow velocity and flow rate which provide a direct functional assessment of the microcirculation. The proposed imaging modality can provide a direct anatomical and functional visualization of microvascular flow of the whole brain *in vivo* and non-invasively at a microscopic scale. These micro-vessels correspond to arteries, arterioles, and pre-capillaries arterioles, which have a fundamental role in the metabolic regulation of cerebral blood flow and are the site of regulation of flow resistance and thus brain perfusion.

**Implications of all the available evidence**

This study should strongly benefit to fundamental research on stroke, aneurysms, brain tumour, vascular cognitive impairment, and neurodegenerative diseases. 3D ULM of mice brain can provide new insights into vascular disease progression, vascular remodelling, tumour growth, efficacy of drugs and other therapeutic interventions, while additionally facilitating the development of biomarkers associated with microcirculatory alterations. In addition, the technology developed in this study is highly translational and has the potential to become a major tool for the clinical investigation of the cerebral microcirculation in various pathologies including for the diagnostic and follow up of stroke, aneurysms, arteriovenous malformation, tumours such as glioblastoma, vascular cognitive impairment and neurodegenerative diseases in human patients.

Alt-text: Unlabelled box

## Introduction

Brain perfusion relies on a complex three-dimensional vascular network of arteries, veins, and capillaries. Alterations of the morphology and/or function of this cerebral vascular network are common features of neurological disorders such as stroke, aneurysms, vascular cognitive impairment, neurodegenerative diseases.^1^, ^2^, ^3^, ^4^, ^5^ Non-invasive high-resolution imaging of the cerebral vascular morphology and function is therefore key for the study of these pathologies. Imaging the vascular networks in the whole brain remains however challenging *in vivo*, as it spans multiple spatial scales (from micron-sized capillaries to millimeter-sized vessels) and multiple scales of velocity (detection from ∼1 mm/s in capillaries to ∼100 mm/s in larger vessels).^6^

Several whole brain imaging techniques have been developed for preclinical cerebrovascular studies. Optical clearing,^7^ micro-CT of vascular cast^8^ or synchrotron-based phase-contrast tomography of perfused brains^9^ approaches have been used to visualize the microvascular brain structures at high resolution in *ex vivo* studies. Micro angiography using MR and CT^10^^,^^11^ revealed images of the vasculature of the whole brain *in vivo* using contrast agents, but these modalities are limited to the visualization of the vessels anatomy without providing the blood flow time dynamics relevant for assessing the cerebrovascular function. Optical imaging has been used to provide blood flow information at the microscopic level but remains limited to the visualization of superficial structures.^12^

The concept of Ultrasound Localization Microscopy was introduced in the early 2010s by analogy to optical super-resolution techniques. The reader can find more details on the early development of ULM in the review article of Christensen-Jeffries et al.^13^ *In vitro* and *in vivo* ULM applications were developed by several groups in the last decade.^14^, ^15^, ^16^, ^17^, ^18^ ULM can map the microvascular flows at high spatial resolution (∼10 µm), an order of magnitude smaller than the ultrasound diffraction limit, and at depths of several centimeters much greater than the traditionally frequency-limited imaging depth. ULM relies on the localization and tracking of single circulating microbubbles in the blood flow. ULM has been applied to the mapping of cerebral microvascular flow in rodents^17^ and in the human brain^19^ using mainly 2D imaging approaches which remain somehow limited to map cerebral vessels that are oriented in all three dimensions. 2D velocity estimates can also be significantly underestimated when the vessels are not perfectly aligned to the 2D slice. Several approaches have been proposed to extend ULM to 3D including 1.5 D arrays,^20^ fully addressed 2D matrix transducers,^21^ multiplexed 2D arrays,^22^ sparse 2D arrays,^23^ hemispherical arrays^24^ and row-column addressed probes.^25^ We introduced a few years ago 3D ultrafast imaging using matrix transducers^26^ and we recently demonstrated 3D ULM in phantoms.^27^

In this study, we demonstrate the feasibility of 3D transcranial ULM imaging of the whole brain in mice. Volumetric acquisitions at ultrafast volume rate were performed through the intact skull while microbubbles were injected into the blood stream. 3D localization and tracking of the microbubbles were performed offline to reconstruct the vascular anatomy and flow, enabling the quantification of vessel size, flow velocity and flow rate at a microscopic resolution.

## Methods

### Ethics

All animals received humane care in compliance with the European Union Directive of 2010 (2010/63/EU), and the study was approved by the institutional and regional committees for animal care (Comité d’éthique pour l'expérimentation animale # 59 - ‘Paris Centre et Sud’, Protocole # 2017–23) and followed the ARRIVE guidelines. The animals were naïve animals, 7-week-old at the experiments time, randomly taken in the cage, none were excluded. The experimental unit in this study is the individual animal.

### Animal preparation

Experiments were carried out in N = 3 C57Bl/6 male mice (Janvier Labs; Le Genest St Isle, France). Animals (three per cage) arrived in the laboratory one week before the beginning of the experiment and were maintained under controlled conditions (22 ± 1°C, 60 ± 10% relative humidity, 12/12h light/dark cycle, food, and water ad libitum).

**Figure 1:**
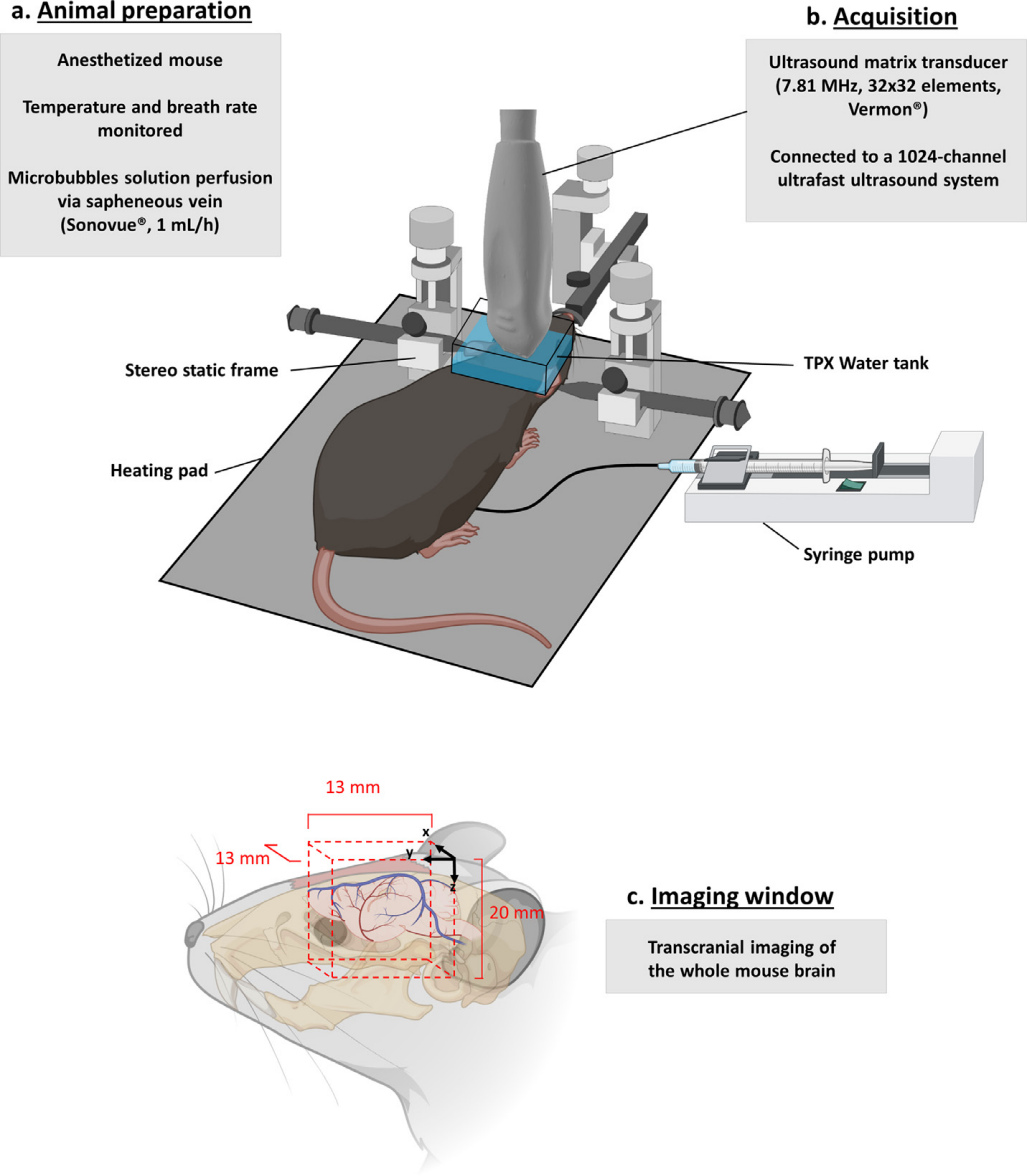
3D transcranial ULM imaging: experimental set up: a. An anesthetized mouse was shaved and placed in a stereotactic frame. A micro bubble solution was perfused continuously via the saphenous vein. b. A 32 × 32 matrix array probe was placed in a water tank on top of the mouse brain without craniotomy. c. A 13 × 13 × 20 mm^3^ volume was imaged covering the whole mouse brain. Figure created with BioRender.com.

At the beginning of the imaging session, the animals were anesthetized using an initial intramuscular (IM) injection of ketamine (Imalgene©, 100 mg.kg−1) and Xylazine (Rompun©, 20 mg.kg−1). The anaesthesia was later maintained by IM infusion of the same aesthetic at the rate of ketamine (33 mg.kg^−1^.h^−1^) and Xylazine (7 mg.kg ^-1^.h-^-1^). Twenty minutes before skin incisions, 100 µL of local aesthetic (Lurocaine (Vetoquinol, France, 7 mg.kg^−1^)) was injected subcutaneously. After skin incision in the inner face of the thigh, the saphenous vein was catheterized using PE10 tube (Braintree Scientific Inc., USA) connected to a 30G needle (Terumo, USA). The animal was then placed in a stereotaxic frame, the skin above the skull was removed. Note: The skull was kept intact. One milliliter of saline solution was gently dropped on the skull, followed by echographic gel (Dexco Médical, France). A small TPX water tank made with a transparent bottom window was placed above the gel and head, allowing the positioning of the 2D matrix array 1.5 cm above desired window of imaging (Figure 1b). This setup was developed to reduce near field artefacts of the matrix probe. This window allowed visualization of a large part of the brain, i.e., between coordinates Bregma +3 mm and Bregma - 6mm.

During all procedures, the animal's body temperature was maintained at 37.0°C using a heating pad (Physitemp, Clifton, USA). Heart and breathing frequencies were constantly measured using ECG, a spirometer from AD Instruments and the ‘Labchart’ software (AD Instruments, Paris, France).

### Microbubbles contrast agent injection

A solution of Sonovue® (Braco, Italy) was prepared using the lyophilized powder blended in 5 mL of saline, as recommended by the manufacturer, and injected by continuous infusion at the rate of 1.5 mL/h using a push syringe (KDS Legato 130, KD Scientific, Holliston, USA). The MB solution was continuously mixed manually during infusion using a magnetic stirrer (two magnetic bars were used: a very small one in the syringe and a large one outside the syringe). The catheter attached to the syringe was short to limit the volume of MB that could not be agitated to 20 µL*.*

Injections only occurred during acquisition times and were stopped between acquisitions. This perfusion method was also compared to bolus injections of 80 µL (see Supplementary Figure 1).

### 3D ultrafast ultrasound imaging

A programmable 1024-channel ultrafast ultrasound system as described in Papadacci et al.^28^ and based on the principle of 3D ultrafast imaging introduced in Provost et al*.*^26^ was used to drive a 32-by-32 matrix-array probe centred at 9 MHz with a 60% bandwidth at -6dB (corresponding to 6.3 – 11.7 MHz), and individual element size of 0.3  ×  0.3 mm^2^ (Vermon, Tours, France). The system was composed of four research electronics (Vantage 256, Verasonics®, Kirkland, WA, USA) which were assembled and synchronized, defining a single device containing 1024-channels in transmission and receive.

**Figure 2:**
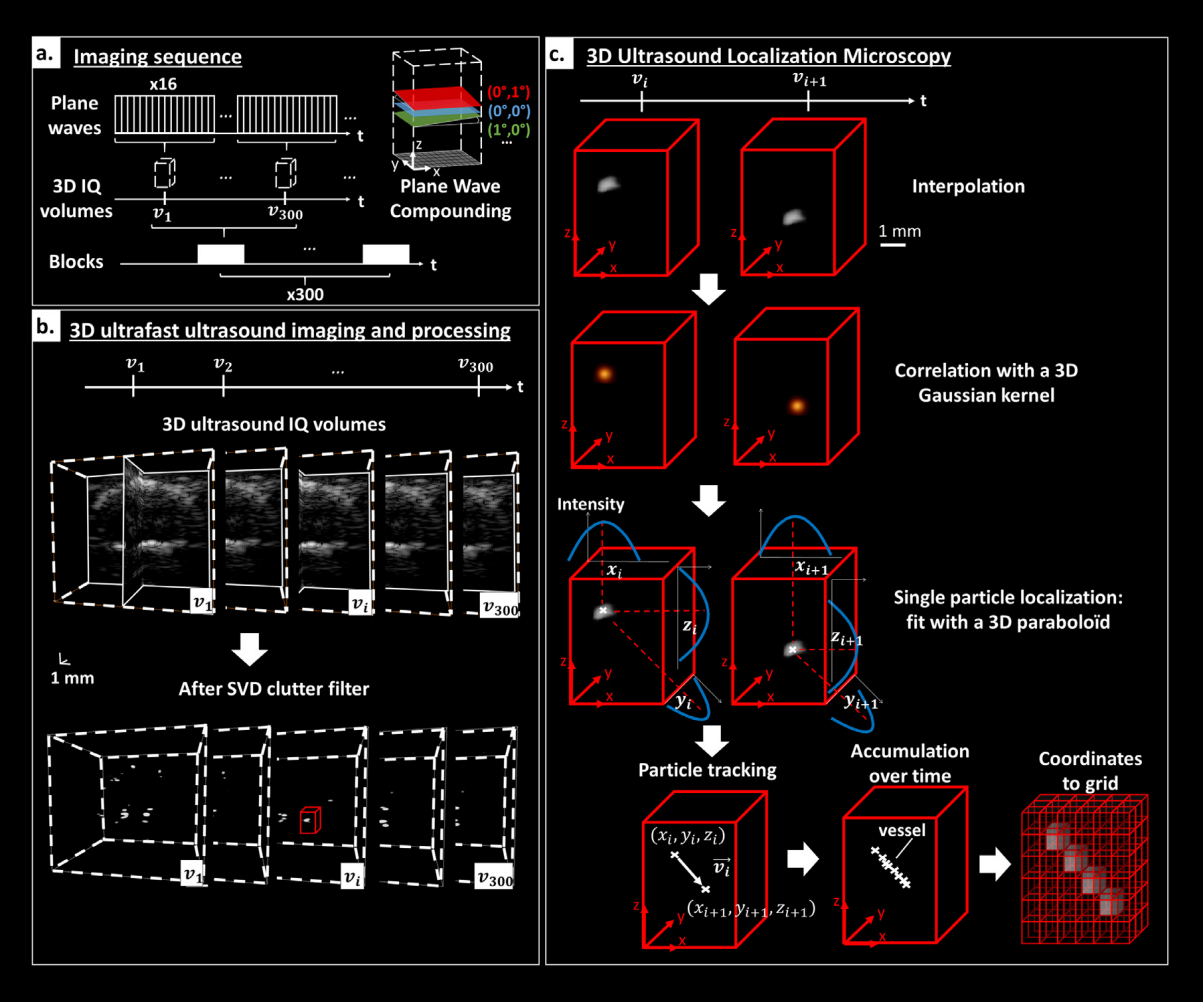
3D Ultrasound Localization Microscopy. a. Imaging sequence: 16 plane waves were compounded to obtain each IQ volume. Blocks of 300 RF were acquired continuously and then written to disk to be beamformed offline. This sequence was repeated 300 times. Examples of tilted plane waves are depicted in the spatial coordinates system of the imaged volume. b. 3D ultrafast ultrasound imaging and processing: After beamforming, IQ volumes were filtered with SVD clutter filter revealing MB flowing through the brain vasculature. A zoom of a MB in the red box is depicted to illustrate 3D ULM processing. c. 3D Ultrasound Localization Microscopy: Volume was first interpolated. The image was then correlated with a 3D Gaussian kernel whose size approaches the size of a MB PSF. In the vicinity of the maximal correlation, a fit with a 3D paraboloid provided the centre position of the particle. Particles were then tracked over time with a Hungarian method and Lagrangian velocities were computed. The accumulation of coordinates was then stored in a 3D grid to compute a density map counting the number of MB in 3D. Velocity components were also stored and gridded to generate 3D velocity maps.

As depicted in Figure 2 an ultrafast Doppler imaging sequence was defined by 16 plane waves [defined by a pair of angles (i,j) with i and j in (-4.2°, - 1.4°, 1.4°, 4.2°)] transmitted at a PRF of 12 kHz giving a volume rate of 750 Hz after coherent compounding. The ultrafast sequence was composed of 300 volumetric images acquired during 400 ms. This sequence was repeated 300 times for an accumulation duration of 120s. A total acquisition duration of 15 min includes breaks of ∼3s between each block of 300 volumetric images for saving and writing on disk. A 2 half-cycle pulse was transmitted with a 100% duty cycle at a frequency of 10 MHz and with a tension of 20 V. The peak rarefaction pressure was measured at 1 MPa, resulting in a mechanical index (MI) of 0.3.

The probe was placed perpendicularly to the surface of the head. To image the brain entirely, the correct placement of the probe was ensured by a four-axis motor module and real-time biplane B-mode imaging.

A 3D delay-and-sum beamforming was performed, as described by Provost et al.,^26^ to reconstruct the ultrasound volumes (i.e., beamformed IQ demodulated data). Volumes of 13  ×  13 ×  24 mm^3^ were reconstructed (lateral directions and depth respectively) with a voxel size of 0.197 ×  0.197 × 0.739 mm^3^. The Beamforming was implemented in CUDA language and performed on GPU units (NVIDIA GeForce RTX 2080 Ti).

Beamformed IQ data were then clutter-filtered using the SVD approach for each sub-block of 150 volumes^29^ and by removing the first 15 eigenvectors. The size of each sub-block was set to 150 volumes for memory limitations.

All the processing was performed with MATLAB software (2020a, The MathWorks Inc., USA) using a 32-core 3 GHz AMD processor. Processing time was approximately 5 h of beamforming and 4 h of SVD filtering plus 3D Ultrasound Localization Microscopy processing for the entire acquisition.

### 3D ultrafast localization microscopy processing

IQ volumes were first interpolated two-fold in each dimension giving a voxel size of 0.1 × 0.1 × 0.04 mm^3^ (lateral directions and depth respectively) and local maxima were then computed over the entire volume.^30^ The local maxima were computed with *imregionalmax* function of Matlab®, which detects connected components of voxels with the same intensity value, surrounded by voxels with a lower value. Each local maximum neighbourhood (kernel of 11 × 11 × 11 voxels) was zero normalized and cross correlated with a Gaussian kernel as in^30^ chosen to approximate a MB Point Spread Function (PSF) whose size was determined using Field II ultrasound simulations (σ_x_ = 0.4 mm, σ_y_ = 0.4 mm and σ_z_ = 0.17 mm). Local maxima whose cross-correlation with PSF exceeded a given threshold (∼0.65) and whose intensity value exceeded a threshold, corresponding to approximately –20 dB, were labelled as micro bubbles.

Small volumes I(x,y,z) (3 × 3 × 3 voxels corresponding to 0.3 × 0.3 × 0.11 mm^3^, lateral directions and depth respectively) centred on these selected local maxima were fitted with polynomials of degree 2 of type $f\left(x,y,z\right)=\;a{\left(x-x_{0}\right)}^{2}+b{\left(y-y_{0}\right)}^{2}+c{\left(z-z_{0}\right)}^{2}$ (Figure 2c). MB centre position were then defined as (x_o_ y_o_ z_o_) for each fit.

MB centre positions were stored over time to be tracked. We used an implementation of the Kuhn-Munkres algorithm or Hungarian method (https://github.com/tinevez/simpletracker, Jean-Yves Tinevez, 2021). For each particle of frame i, the square distance to all particles in frame i+1 was computed. The optimal pairing was given by minimizing the total square distance of matched particles. This algorithm was then looped in every frame. A maximum distance of 0.13 mm (corresponding to a maximum velocity of 100 mm/s) between two subsequent positions was allowed. Tracks smaller than 4 frames (∼5 ms) were also rejected.

The tracks were then used to compute velocity measurement in three dimensions, as explained in Heiles et al.^27^ Each particle had its instantaneous velocity computed, as the ratio between its displacement vector and its temporal sampling. Traditional Lagrangian approach was used to calculate velocity fields.

Eventually, density maps were reconstructed from the MB centre positions. Providing a voxel size, the intensity value was given by the number of detected MB in this voxel. The MB trajectory was then interpolated at a sampling of 20 µm to improve the reconstructed maps. Similarly, velocity maps were reconstructed from the amplitude velocity of every MB or one of their velocity components along the x, y, or z axis. The velocity value in each voxel was taken as the average velocity measured for each MB passing through this voxel. These velocity maps lead to quantitative and localized maps of brain blood flow velocity.

### 3D motion correction

A 3D motion correction was used to compensate small motion drifts of the animal head (about 20µm/min) that occurred during the acquisitions. To compensate small motion between blocks of acquisition, MB centre position was registered using an iterative closest point (ICP) algorithm. The rigid transformation that minimized the Euclidean distance between the clouds of MB positions tracked for 20 blocks of 150 volumes and the cloud of MB positions of the first 20 blocks was computed. Resulted ICP transformations were applied to obtain motion corrected MB positions.

### Automatic 3D registration to Allen atlas

The geometrical transformation was automatically estimated using Iconeus Brain Positioning System solution described in Nouhoum et al^31^ and implemented in IcoStudio software (Iconeus, Paris, France). Briefly, an iterative intensity-based registration algorithm (maximizing mutual information) allowed to find the optimal affine transformation aligning a Power Doppler volume (20 first seconds of the ULM continuous acquisition accumulation) to the Iconeus mouse brain Doppler angiography reference, already prealigned with the Allen Atlas. Then, this transformation could be applied to any volumetric map generated from the microbubbles tracks and used to extract MB tracks from any regions of interest for quantification.

### 3D visualization

3D representations of vessels were rendered using the *volren* function of Amira software (Amira v.6.0.1 software, Visualization Sciences Group, USA), and IcoStudio software (IcoStudio v1.0.1 software, Iconeus, France).

### 3D quantification

#### Diameter quantification through skeletonization

Quantification of vessel diameter was performed on the 3D density maps reconstructed with isotropic voxels of size 20 µm. In Amira software, a Gaussian filter was applied (sigma = 40 µm and kernel of 3 × 3 × 3 voxels) as well as the “auto-skeleton” function computing a skeleton where density value exceeds 1. Radius was then computed for every centreline point in all detected vessels.

#### Flow rate quantification

The amplitude of the flow velocity was measured at the centreline points of the skeleton and assumed to be the maximum velocity of the velocity profile. Using the velocity and diameter measured previously, the flow rate was eventually computed assuming a Poiseuille flow: $Q=\frac{V_{max}}{2}\pi R^{2}$ (Q: flow rate, V_max_: maximum velocity, R: radius) for every detected vessel.

#### Allen-based MB spatial density and MB velocity estimates

Thanks to the affine geometrical transformation aligning the Allen atlas and the ULM volume, the coordinates of any anatomical region contours could be transformed in the ULM acquisition space, allowing to select the MB tracking points contained in this region. Then, we were able to estimate and compare the spatial MB density or the MB velocity distributions between different anatomical regions. The MB spatial density in MB counts per mm^3^ could be computed by normalizing the MB count by the considered volume of brain region in mm^3^. The MB velocity distribution was automatically obtained by deriving the instantaneous velocity along the points of each track contained in the segmented region.

#### Spatial resolution

A density map was reconstructed with isotropic voxels of 10 µm. We would like to clarify here that 10 × 10 × 10 µm^3^ is not the kernel for localization but is the voxel size of the 3D density map reconstructed from all the MB coordinates. Vessels were isolated and an intensity profile was extracted from a cross section of 10 µm. Points were sampled with a distance step of 10 µm as depicted in Supplementary Figure 3.B.iii and full width at half maximum (FWHM) was measured.

#### MB velocity estimation in different layers of the PPC

Using the geometrical transformation found automatically between the mouse brain vasculature and the Allen atlas template, MB tracks contained in each layer of the PPC could be extracted. Then, MB instantaneous velocity distributions in venules (negative) and arterioles (positive) were computed for each PPC sublayers, by computing the amplitude of the instantaneous MB velocity at each tracking position.

### Statistics

All statistical analyses were performed using MATLAB. This proof-of-concept study was designed to show the feasibility of the technique and there was no need to determine an optimal number of animals. Comparisons of the MB velocity distributions in the microvessels among different Posterior Parietal Cortex (PPC) sub-layers (from layer 1 to layer 6a) was performed in one representative mouse. Distributions were analysed using an unpaired two-sample Student t-test. A *p* value (two-sample t-test) less than 0.05 was considered statistically significant. All the MB velocity distributions associated with each layer were detected as normal using the Anderson-Darling test.

### Data availability

MB density maps and MB velocity maps are available at a data repository (https://doi.org/10.5281/zenodo.6328308). Other data that support the findings of this study are available from the corresponding author upon reasonable request. Researchers wishing to obtain the raw data must contact the Office of Research Contracts at INSERM to initiate a discussion on the proposed data transfer or use.

### Role of funders

The funders had no role in study design, data collection, data analyses, interpretation, or writing of the manuscript. The corresponding author (M. Pernot) had full access to all the data and the final responsibility for the decision to submit for publication.

## Results

### Whole brain mapping of the vascular anatomy

**Figure 3:**
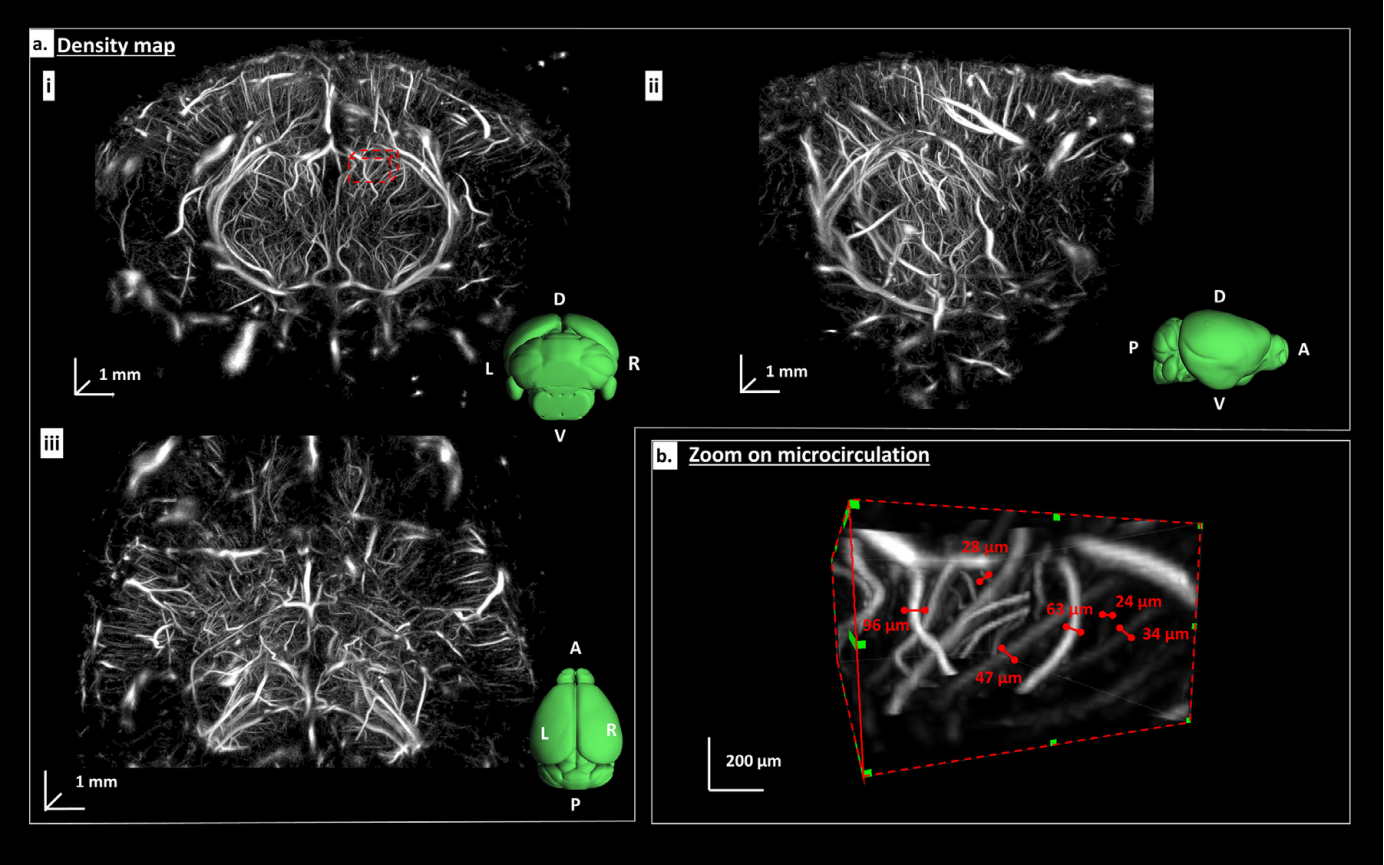
*In vivo* transcranial 3D ULM of the mouse brain vasculature: a. Density map of a representative mice brain in a coronal (i), sagittal (ii) and top (iii) view. An Allen atlas of the mice brain is depicted in green with its orientation (L: Left R: Right A: anterior, P: Posterior, D: Dorsal, V: Ventral). Grayscale values are proportional to the number of micro bubbles detected. b. Zoom of micro vessels in a region depicted by a red-dashed box in A. The diameter of microvascular vessel is measured with a 3D calliper at FWHM.

3D ULM provides high resolution images of the whole brain vasculature as shown on Figure 3a. A total of 3 × 10^6^ MB were detected and tracked by the algorithm during the acquisition. 3D renderings of coronal (Figure 3a.i), sagittal (Figure 3a.ii) and top (Figure 3a.iii) views shows the complex vascular architecture of the brain. When zooming at a smaller scale, the microcirculation can be visualized, as displayed in Figure 3b. In addition to large vessels (∼100µm diameter), blood vessels as small as 20 µm in diameter can be visualized (Figure 3b and Supplementary Figure 3.B). For comparison, the low-resolution 3D power doppler is provided in Supplementary Figure 2.

### Flow assessment

**Figure 4:**
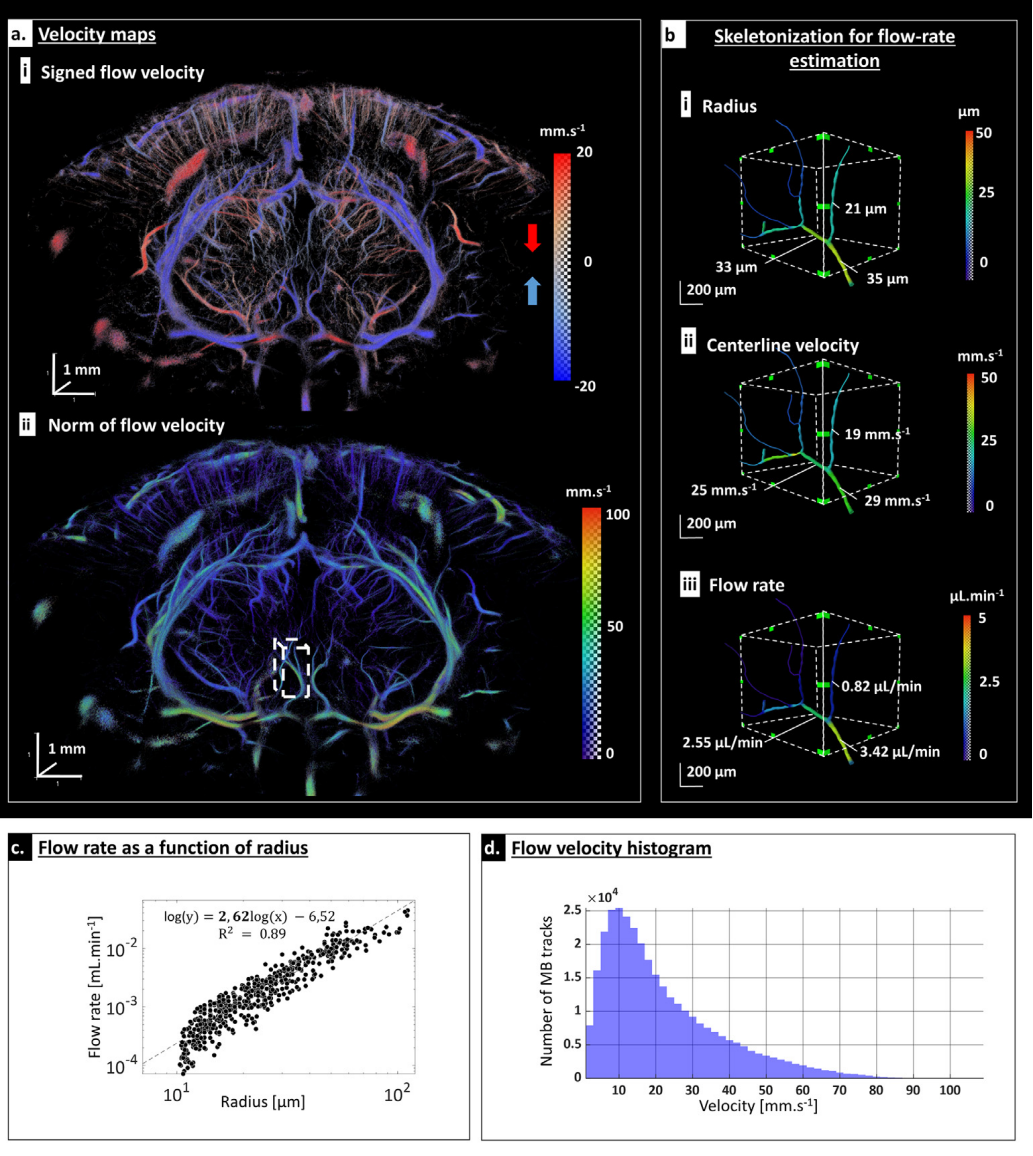
Multi scale flow velocity quantification: a. Velocity map. In (i) velocity is signed along depth direction revealing upward (blue) and downward (red) flow. In (ii) the amplitude of the velocity field is depicted. b. Flow rate quantification illustrated in a small region (white-dashed box in A.ii): after skeletonization, the radius is automatically estimated in all vessels (i). Flow velocity is then computed along the centreline points (ii) and flow rate is then measured for each segment assuming a Poiseuille flow (iii). c. Analysis of flow rate as a function of radius in a large range of vessels reveals a power law with an exponent of 2.62. X and Y axis are in log scale. d. Flow velocity distribution of MB.

The high temporal resolution of the 3D ultrafast imaging system further enables estimating the flow velocity via particle tracking. Figure 4a.i shows the dorsal to ventral flow direction: downwards (in red) and upwards (in blue). Flow velocity estimates range from 2 mm/s in arterioles and venules up to 100 mm/s in large arteries as shown in Figure 4a and b. Figure 4d shows the flow velocity distribution across the entire brain from a few mm/s for small arterioles and venules up to 100 mm/s for larger branches. Moreover, it was possible to accurately assess the velocity profile across a relatively large vessel, revealing a Poiseuille-like flow profile (Supplementary Figure 3c).

Quantification of the vessel diameter is also performed automatically, using a 3D centreline extraction (skeletonization) algorithm (skeleton, Amira v.6.0.1 software, Visualization Sciences Group, USA). Diameters range from 20 µm for arterioles and venules to ∼200 µm for larger branches. In these segmented vessels, the flow velocity is also automatically measured at the centreline points. Assuming a Poiseuille flow, flow rates ranging from ∼0.1 µL/min to 50 µL/min are derived (Figure 4b).

The flow rate / radius relation is also obtained for a wide range or radius (Figure 4c). Fitting to a power law provides an exponent of 2.62 (r² = 0.89), which is consistent with theoretical predictions and experimental validations of scaling laws in vascular trees, also known as the generalized extension of Murray's law [6].

### Automatic atlas registration and Allen based quantification

#### Automatic atlas registration

**Figure 5:**
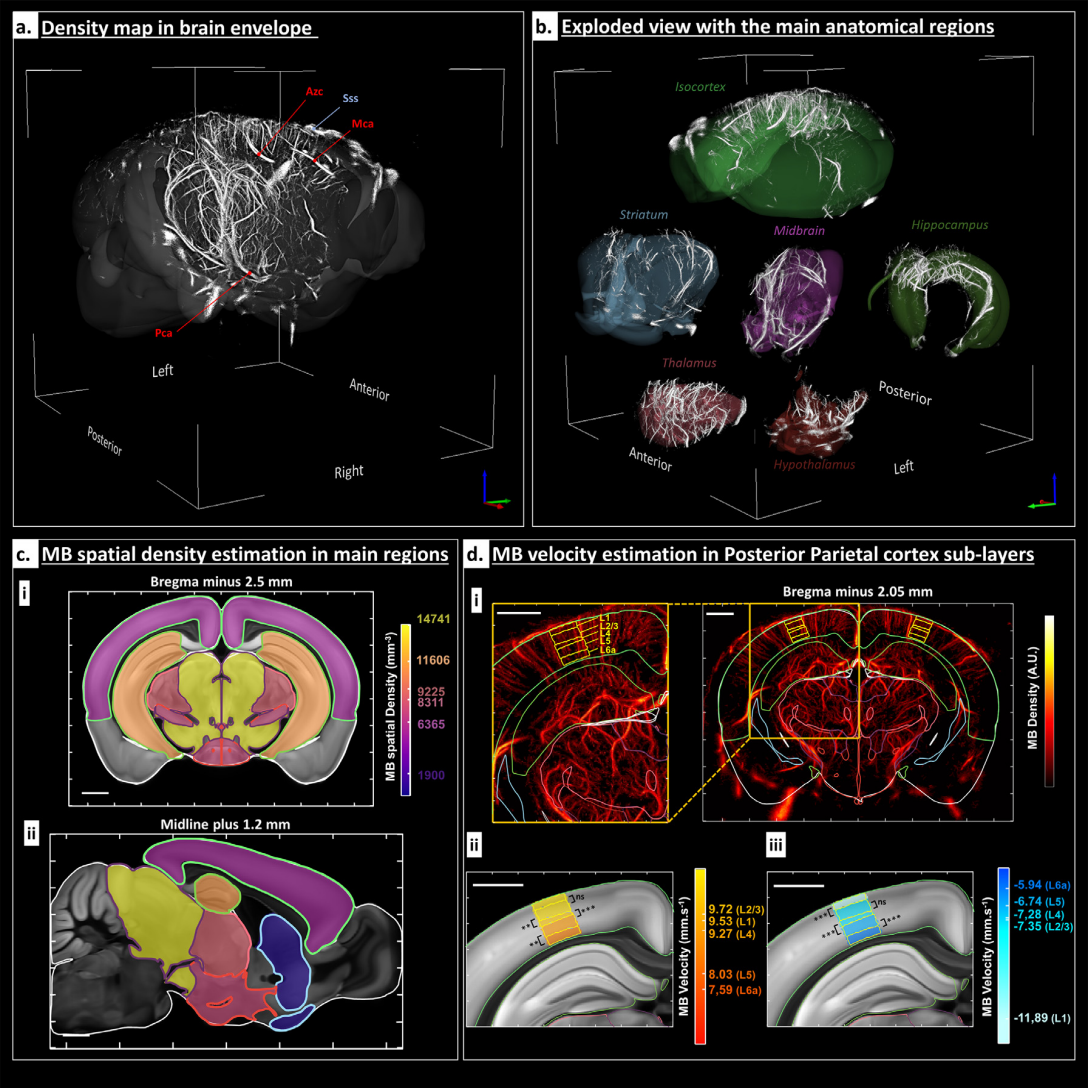
Atlas based registration and its use for quantifications: a. The MB density map was aligned with the Allen mouse brain atlas. The main arteries are labelled: Pca (Posterior Cerebral Artery), Mca (Medial Cerebral Artery) and Azc (Azygos Anterior Cerebral Artery). In blue, the Superior Sagittal Sinus (Sss) is also indicated. b. Exploded view with six anatomical regions: Isocortex, Hippocampus, Midbrain, Striatum, Thalamus and Hypothalamus. c. Average MB spatial density in the main cerebral regions. The average MB spatial density was computed in each of the main region presented in B as a measure of the average flow rate in these regions. Results are represented on a coronal slice at Bregma minus 2.5 mm (i) and a sagittal slice at the midline (sagittal suture) plus 1.2 mm (ii) intersecting all these regions. Main regions edges are marked with the same colours as in the Figure 5.B. The spatial density values for each region in MB.mm^−3^ are superimposed with the Allen two-photon template. Scalebars: 1 mm. d. MB velocity estimation in the Posterior Parietal cortex (PPC) anterior area sub-layers. The results are represented on a coronal slice at Bregma minus 2.05 mm, represented at first through a MIP (1.5 mm thickness) in (i) right. PPC layers from 1 to 6a are delimitated in yellow, see the legend from the zoom shown in (i) left. In D.ii and D.iii, the colour overlayed to the Allen two-photon template indicates for each PPC layers the mean of the MB velocity distribution for arterioles (ii) and venules (iii). All the MB velocity distributions associated with each layer were detected as normal with Anderson-Darling test. Significant differences between the mean MB velocities were calculated using the following p-values (two-samples Student *t*-test); *P < 0.05, **P < 0.01, ***P < 0.001. Scalebars 1 mm.

As previously described, the affine transformation is estimated and applied to the volumetric MB density map (voxel size = 20 × 20 × 20 µm^3^). The resulting volume is superimposed to the Allen mouse brain atlas. As it can be seen in Figure 5a, the field of view allows visualizing the microvasculature from the end of the cerebellum until the beginning of the olfactory bulb (from Bregma – 6 mm to Bregma plus 3 mm), and the main cerebral vascular vessels can be identified (see legends in Figure 5a). Binary masks corresponding to six different regions of the brain (Isocortex, Hippocampus, Midbrain, Striatum, Thalamus and Hypothalamus) are computed and applied to the same MB density map, allowing to split and represent the micro vascularization for each region (Figure 5b).

#### Flow Quantification

The MB spatial density was computed in the different regions of the brain as a measure of the average flow rate in the region. Results are shown in Figure 5c, as an overlay on the two-photon Allen template (25 × 25 × 25 µm^3^), in a coronal slice and a sagittal slice.

To go further in the flow analysis, we compared MB velocity in the microvessels of different Posterior Parietal Cortex (PPC) sub-layers (from layer 1 to layer 6a). For each layer, arterioles and venules velocity distributions were separated regarding the sign of the velocity along z axis (positive for arterioles and negative for venules). As shown in Figure 5d, arterioles showed higher MB velocities than venules, excepted in layer 1 (the average velocity in all sub-cortical layers is 8.8 mm.s^−1^ for arterioles versus -7.8 mm.s^−1^ for venules). The velocity was found to decrease from superficial layers to deeper layers (starting from L2/3 for arterioles and L1 for venules).

## Discussion

We hereby demonstrated *in vivo* transcranial 3D ULM of the mice brain. Imaging of cerebral microvascular vessels was achieved over the whole brain and across a large range of vessels from 20 to 200 µm and flow velocities from ∼2 to 100 mm/s. Apart from its fine spatial resolution and mapping of the whole cerebral vasculature, 3D ULM enables quantification of flow velocity, vessel diameter and flow rate.

This proposed imaging modality provides a direct anatomical and functional visualization of microvascular flow of the whole brain *in vivo* and non-invasively at a microscopic scale. These micro vessels correspond to arteries, arterioles, and pre-capillaries arterioles, which have a fundamental role in the metabolic regulation of cerebral blood flow and are the site of regulation of flow resistance and thus brain perfusion.^32^^,^^33^

In this work, we automatically registered the brain ULM microvasculature volumes to a reference vascular dataset aligned with the Allen Brain Atlas which can allow to trace up to 300 anatomical brain regions^34^ automatically. It allowed extracting per-regions microvascular parameters such as MB density and velocity, but we could also extract diameters and flow rate. By investigating six main large regions, we found that the MB spatial density ranged from 1900 MB.mm^−3^ in the striatum to 14741 MB.mm^−3^ in the midbrain. We were also able to characterize MB velocities for both arterioles and venules located in the PPC and how this velocity decreases over deep sub-cortical layers. Such quantifications could then be used for high-throughput microvascular phenotyping of genetic mice models, longitudinal monitoring of drug effects on the vasculature or correlation with gene expressions atlas.^35^

In a broader perspective, 3D ULM of mice brain should strongly benefit to fundamental research on stroke, aneurysms, brain tumour, vascular cognitive impairment, and neurodegenerative diseases. 3D ULM of mice brain can provide new insights into vascular disease progression, vascular remodelling, tumour growth, efficacy of drugs and other therapeutic interventions, while additionally facilitating the development of biomarkers associated with microcirculatory alterations.

The technology developed in this study is highly translational and has the potential to become a major tool for the clinical investigation of the cerebral microcirculation in various pathologies including for the diagnostic and follow up of stroke, aneurysms, arteriovenous malformation, tumours such as glioblastoma, vascular cognitive impairment and neurodegenerative diseases. The technology developed in this work could have a strong clinical impact for the diagnostic and monitoring of vascular diseases and microcirculation alterations. The strength of this approach is the capability to directly visualize the microvascular flow which is not possible with other modalities because of their limited spatial resolution. In addition, the technology could be used repeatedly at the bedside of patients to monitor the evolution of vascular diseases, which remains complex to perform with existing imaging modalities because of complex access (MRI) or radiation risks (CT). We recently demonstrated a first *in vivo* proof of concept of 2D ULM for imaging the vasculature of the human brain using a conventional ultrasound transducer.^19^ Translation to 3D ULM of the human brain will require the use of matrix transducers at a lower frequency between 1 and 3 MHz. 3D Ultrafast imaging with a large field of view has been recently demonstrated for cardiac applications using matrix transducers with diverging wave emissions at 2.5MHz^36^, ^37^, ^38^ and this approach could be translated to 3D ultrafast imaging of the human brain through the temporal window. Imaging of the human whole-brain will however require the development of dedicated large aperture matrix transducers. Ultrasonic wave aberrations induced during the propagation through the human skull bone have long been considered as a major issue for transcranial ultrasound brain imaging. In this study, we did not apply any correction of the aberrations induced by the propagation of the ultrasonic wave through the skull bone as most of the MB were localized efficiently without any aberration correction. Although the mouse skull is relatively thin, significant phase and amplitude aberrations could be induced at several locations of the skull bone which may affect the vessel reconstruction and the flow quantification underneath these locations. Aberration correction techniques such as the one used in Demene et al^19^ could further improve the MB detection and localization accuracy.

Like any other imaging modalities, 3D ULM has limitations. First, it requires acquisitions of several tens of seconds and the processing takes several hours. Recently, deep learning based algorithms showed a great potential, shortening the MB localization processing time and performances.^39^^,^^40^ As it helps to distinguish overlapping PSFs centroids, higher MB concentrations could be handled, thus shortening the time of acquisition. Second, the current spatial resolution, estimated at 20 × 20 × 20 μm^3^ could be further improved by using matrix arrays at higher central frequency but at the expense of a lower Signal to Noise Ratio (SNR) because of the attenuation and ultrasonic wave aberrations due to the skull bone. The use of diverging waves sequences and the development of dedicated matrix probes with smaller spatial pitch could help to further increase the field of view. Finally, even though 1024 channel acquisition systems equivalent to the one used in the study are commercially available, the availability in research labs remains limited by its cost and cumbersomeness. The 1024 channel system could be replaced by a single 256 channels system combined with electronic multiplexors, as it was recently demonstrated on the rat brain.^22^ This approach, however, requires longer acquisition times and more importantly induces a significant loss of the temporal resolution. In this last study,^22^ the use of 4:1 multiplexors reduced the volume rate by ten times. Such a strong imaging rate reduction affects the quality of velocity estimates in particular in large vessels. High imaging rates is indeed critical for accurate velocity estimation of rapid flow (>70 mm/s). Row-column addressing approaches^25^^,^^41^ could also be an interesting alternative, offering wider field of views although it remains to be seen if this strategy can reach the high-volume rate required to properly quantify blood velocities at the different scales involved.

We have demonstrated in this work, 3D ultrafast localization microscopy of the intact mouse brain *in vivo*. Using a 1024 channels ultrafast ultrasound scanner, we could image and track individually microbubbles circulating in blood vessels. We could map vessels down to 20 micrometers and assess associated hemodynamic parameters locally or in any brain regions thanks to the Allen brain atlas. 3D brain ULM of mice models could provide new insights into vascular disease progression, vascular remodelling, tumour growth, efficacy of drugs and other therapeutic interventions at unprecedented vascular scale for an *in vivo* technique.

## Contributors

M.P., T.D., B.O, S.P, and M.T conceived the study. O.D., C.P., and M.P. developed sequence acquisitions, O.D., A.B., C.P., S.P., N.I. acquired data. O.D., A.B., T.D., M.T and M.P. performed data processing. O.D., A.B, S.P., C.P, M.T. and M.P. interpreted the results. M.P, T.D., A.B. and O.D. wrote the first draft of the manuscript with substantial contribution from S.P, M.T and B.O. All authors edited and approved the final version of the manuscript. Underlying data have been verified by M.P and T.D.

## Declaration of interests

M.T, M.P, B.O and T.D are co-founders and stockholders of Iconeus and have received fundings from Iconeus for research on functional ultrasound imaging. B.O and A.B are employees of Iconeus.

## Appendix Supplementary materials

Image, application 1

Image, application 2
