# Supplementary material for: In vivo whole brain microvascular imaging in mice using transcranial 3D Ultrasound Localization Microscopy
Source: eBioMedicine. 2022 Apr 20;79:103995. doi: 10.1016/j.ebiom.2022.103995 (PMC9048085; doi:10.1016/j.ebiom.2022.103995)
Supplement: Supplementary file 2 [file mmc2.docx]

1. Kindly provide a word file named “caption for supplementary material” with the list of captions for each Supplementary file as they should appear online.

**Supplementary figure 1. Number of microbubbles (MB) tracked using Bolus versus continuous injection: a.** Evolution of the number of MB tracked after injection of an 80 µL bolus. The red dashed line indicates the time corresponding to the beginning of the injection. The maximum of tracked MB is reached after less than 2 minutes. **b.** When injecting continuously, the number of tracked MB reaches a plateau after 5 minutes.

**Supplementary figure 2. Comparison of resolution: a. Density map** of a representative mice brain in a coronal view. An Allen atlas of the mice brain is depicted in green with its orientation (L: Left R: Right A: anterior, P: Posterior, D: Dorsal, V: Ventral). Grayscale values are proportional to the number of micro bubbles detected. **b.** For comparison, the **power Doppler** Volume is shown (Voxel size = 197 x 197 x 74 µm^3^) with lower resolution.

**Supplementary figure 3. Density and velocity profiles: a. Coronal view** with a 3D density map (i) and a velocity map (ii). **b. Zoom of micro vessels in a cortical region** depicted by a red-dashed box in (A.i) is given in 3D (i). Two vessels are identified. A 2D slice provided a cross section view of the two vessels (ii). The intensity profile (iii) was computed along the line with steps of 10 µm depicted with dots. Analysis of the profile produced a full width at half maximum (FWHM) of 20 and 30 µm for vessel 1 and 2 respectively. **c. Velocity profile is described from a vessel** identified in a white-dashed box in (A.ii). A 3D view (i) and a 2D cross section (ii) are given. A 1D velocity profile (iii) was computed along the line in the cross section. Flow velocities were estimated along the line with a step of 10 µm and are depicted with dots on the profile. Analysis of the velocity profile in this vessel (FWHM of 70 µm) shows the variations of flow velocity across the vessel and the Poiseuille-like flow profile.
